# Unraveling cancer progression pathways and phytochemical therapeutic strategies for its management

**DOI:** 10.3389/fphar.2024.1414790

**Published:** 2024-08-23

**Authors:** Vikas Sharma, Anis Ahmad Chaudhary, Sweta Bawari, Saurabh Gupta, Richa Mishra, Salah-Ud-Din Khan, Mohamed A. M. Ali, Mohammad Shahid, Saurabh Srivastava, Devvrat Verma, Arti Gupta, Sanjay Kumar, Sandeep Kumar

**Affiliations:** ^1^ Metro College of Health Sciences and Research, Greater Noida, India; ^2^ School of Pharmacy, Sharda University, Greater Noida, India; ^3^ Department of Biology, College of Science, Imam Mohammad Ibn Saud Islamic University (IMSIU), Riyadh, Saudi Arabia; ^4^ Amity Institute of Pharmacy, Amity University, Noida, India; ^5^ Department of Biotechnology, GLA University, Mathura, India; ^6^ Department of Computer Engineering, Parul University, Vadodara, India; ^7^ Department of Biochemistry, College of Medicine, Imam Mohammad Ibn Saud Islamic University (IMSIU), Riyadh, Saudi Arabia; ^8^ Department of Biology, College of Science, Imam Mohammad Ibn Saud Islamic University, Riyadh, Saudi Arabia; ^9^ Department of Biochemistry, Faculty of Science, Ain Shams University, Cairo, Egypt; ^10^ Department of Basic Medical Sciences, College of Medicine, Prince Sattam bin Abdulaziz University, Al-Kharj, Saudi Arabia; ^11^ School of Pharmacy, KPJ Healthcare University, Nilai, Malaysia; ^12^ Department of Biotechnology, Graphic Era (Deemed to be University), Dehradun, Uttarakhand, India; ^13^ Lloyd School of Pharmacy, Greater Noida, India; ^14^ Biological and Bio-computational Laboratory, Department of Life Science, Sharda School of Basic Sciences and Research, Sharda University, Greater Noida, India; ^15^ DST-FIST Laboratory, Department of Life Sciences, School of Basic Sciences and Research, Sharda University, Greater Noida, India

**Keywords:** cancer prevention, molecular pathways, effector molecules, herbal metabolites, toxicology

## Abstract

Cancer prevention is currently envisioned as a molecular-based approach to prevent carcinogenesis in pre-cancerous stages, i.e., dysplasia and carcinoma *in situ*. Cancer is the second-leading cause of mortality worldwide, and a more than 61% increase is expected by 2040. A detailed exploration of cancer progression pathways, including the NF-kβ signaling pathway, Wnt-B catenin signaling pathway, JAK-STAT pathway, TNF-α-mediated pathway, MAPK/mTOR pathway, and apoptotic and angiogenic pathways and effector molecules involved in cancer development, has been discussed in the manuscript. Critical evaluation of these effector molecules through molecular approaches using phytomolecules can intersect cancer formation and its metastasis. Manipulation of effector molecules like NF-kβ, SOCS, β-catenin, BAX, BAK, VEGF, STAT, Bcl2, p53, caspases, and CDKs has played an important role in inhibiting tumor growth and its spread. Plant-derived secondary metabolites obtained from natural sources have been extensively studied for their cancer-preventing potential in the last few decades. Eugenol, anethole, capsaicin, sanguinarine, EGCG, 6-gingerol, and resveratrol are some examples of such interesting lead molecules and are mentioned in the manuscript. This work is an attempt to put forward a comprehensive approach to understanding cancer progression pathways and their management using effector herbal molecules. The role of different plant metabolites and their chronic toxicity profiling in modulating cancer development pathways has also been highlighted.

## Graphical Abstract

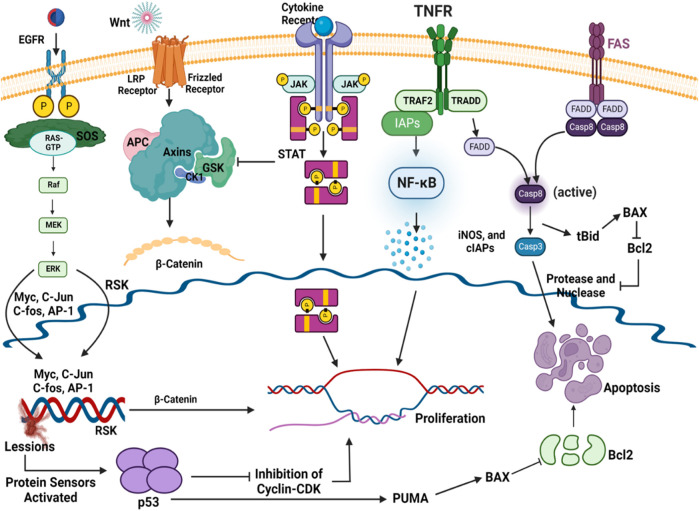

## Highlights

• Molecular investigation of pre-cancerous lesion development in cancer progression pathways is identified as a potential target in cancer prevention.
• Modulation in the expression of effector molecules like SOCS, p53, VEGF, STAT, BAX, Bcl2, NF-κB, CDKs, and β-catenin may provide an opportunity to hamper the carcinogenic continuum at different stages of progression.
• Phytochemicals like 6-gingerol, silymarin, resveratrol, apigenin, EGCG, and β-carotene impair signaling pathways and inhibit the development of cancer *in situ*, thereby preventing the progression to invasive cancer.

## 1 Introduction

Cancer is one of the leading causes of death around the globe. Continuous exposure to risk factors, including physiological, behavioral, environmental, and professional factors, is attributed to the increase in cancer burden worldwide (Eloranta et al., 2021; Ferlay et al., 2021). Approximately 90% of cancer cases are an outcome of environmental factors and somatic mutations, and the rest are due to mutations in the germline (Sung et al., 2021).

**FIGURE 1:**
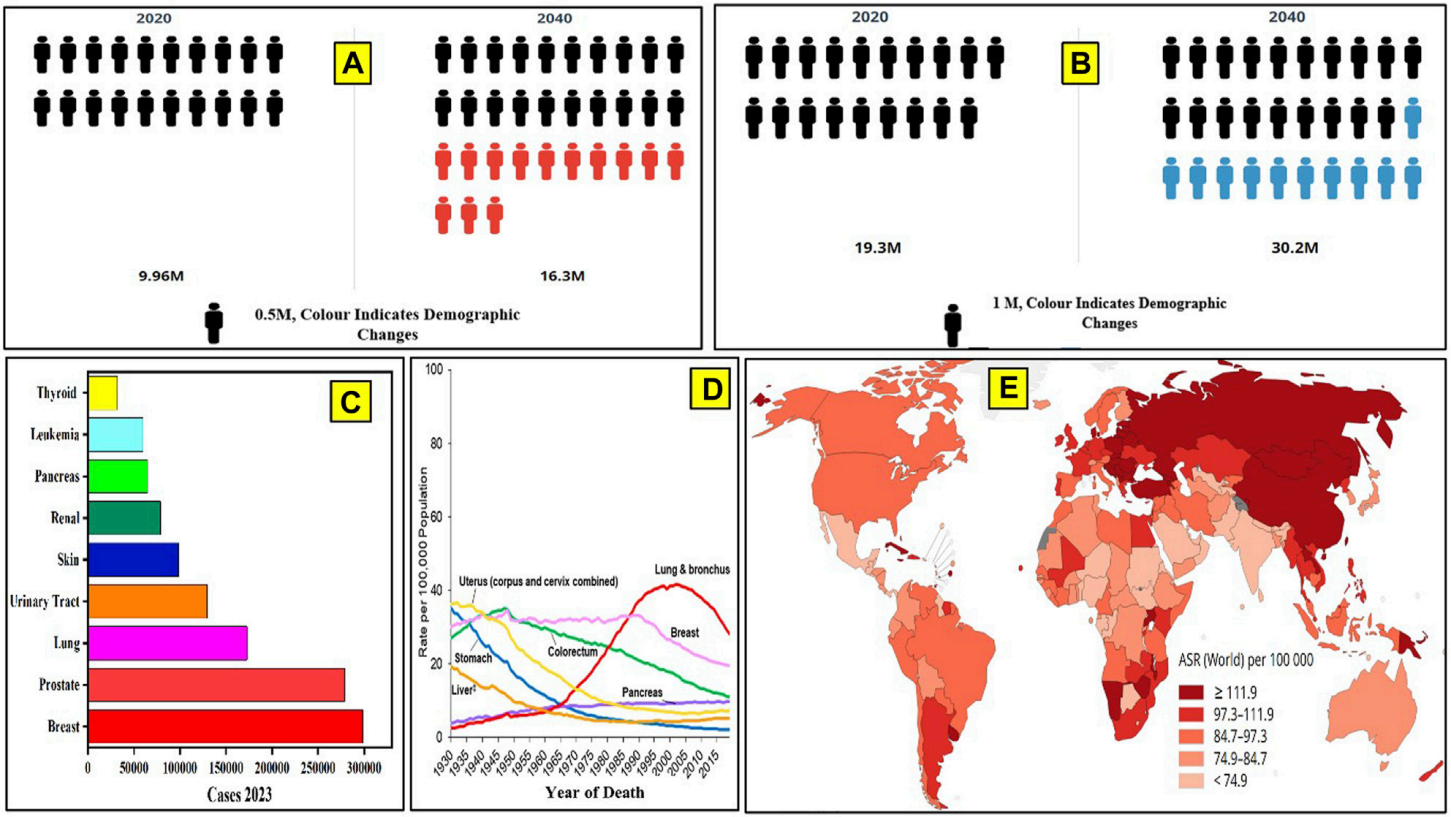
Global cancer data. **(A)** Number of cancer cases in 2020 and the cases projected by 2040, as per WHO. **(B)** Number of deaths due to cancer reported until 2020 and the number of deaths projected up to the year 2040. **(C)** Number of cancer cases reported in the United States in 2023. **(D)** Cancer mortality data from 1930 to 2020. **(E)** Number of cancer-related deaths in different continents in a calendar year (2020).

According to the World Health Organization (WHO) and the Global Cancer Observatory (GCO), more than 19 million people are currently affected by cancer, and the number of active cases may rise to more than 28 million people by 2040 in a calendar year. Cancer mortality data have been analyzed by the WHO and GCO from 1930 to 2020 (Mathur et al., 2020; Siegel et al., 2020). Lung and bronchus cancer have a higher mortality rate, followed by breast, uterus, and stomach cancer (Mathur et al., 2020; Siegel et al., 2020). Figure 1 depicts the number of cancer-afflicted patients and the mortality reported in recent databases and projections up to 2040. Prostate cancer has been reported to be the most common cancer among males (29%), and breast cancer (31%) is most common among females (Figure 1C). Cancer death rates have been declining in males and females since the early 1990s; from 1991 to 2019, the combined death rate dropped by 32%. Breast cancer death rates changed little between 1930 and 1989 but decreased by 42% from 1989 to 2019 due to earlier detection and improved treatment. It is indicated that there has been a significant drop in the mortality rates for a few specific cancers, like stomach and colorectal cancer, in recent years. (Figure 1D). However, the predicted new cancer cases per year in Asia and Africa are expected to rise to 59% and 89% of the current prevalence, respectively, by 2040 (Jones and Baylin, 2007).

**FIGURE 2:**
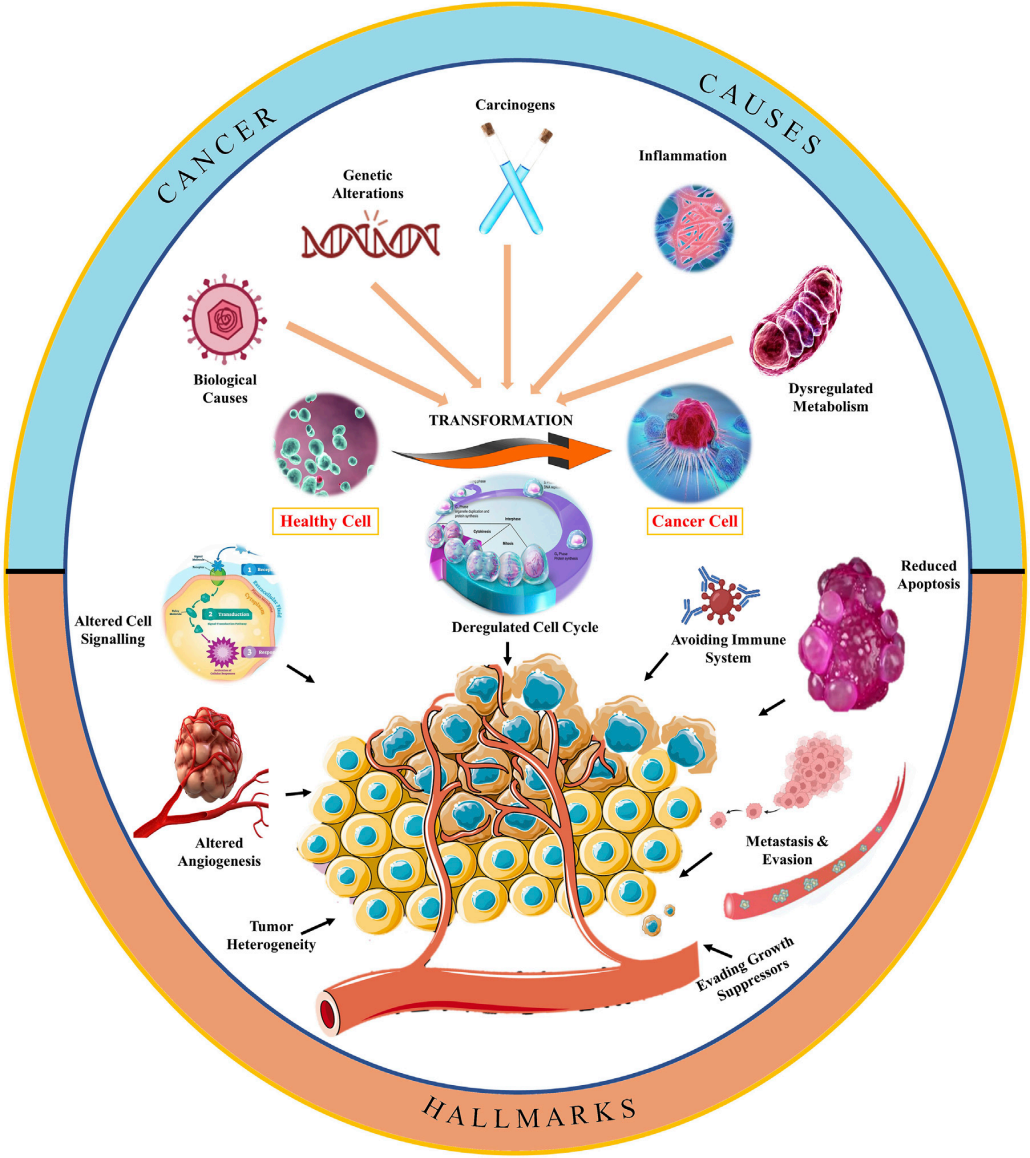
Schematic representation of hallmarks of oncogenesis.

Cancer progression is a complex biological process with a diversified understanding (Jones and Baylin, 2007). However, different pathways have been extensively studied in the last few decades due to advancements in imagining and analysis technology (Arruebo et al., 2011). The general landscape of oncogenesis has been depicted in Figure 2 which represents several risk factors that convert normal healthy cells into abnormal cancerous cells through molecular manifestations (Wang et al., 2017). Carcinogens, biological agents, and inflammation may trigger deregulated metabolism, which leads to genetic mutation and may result in the development of cancer (Gandhi et al., 2014). These manifestations at the cellular level may start interfering at cell cycle checkpoints (Liu C. G. et al., 2020). Figure 2 depicts that the altered cell cycle affects cell signaling and also starts avoiding immune cells (Zheng and Ma, 2017). A series of biological events are the reason for the transformation of a healthy cell into a cancerous cell, including carcinogens, genetic alterations, inflammation, and dysregulated metabolism. At the molecular level, such factors of cancer development disbalance the levels of tumor suppressor genes and oncogenes, resulting in cell cycle deregulation. Furthermore, replicative immortality and altered cell signaling mechanisms are causing the progression of cancer and have been reported to avoid immune cells, growth suppressors, and angiogenesis, which yields the metastasis of cancerous cells. The role of cell death resistance and uninterrupted angiogenesis has also been linked to cell proliferation and cancer development (Figure 2).

Nowadays, chemotherapy, targeted therapy, radiotherapy, and surgical techniques are commonly employed in cancer treatment interventions. However, each of the conventional treatment options possesses its inherent challenges that increase the chances of low quality of life for cancer patients, including side effects and the peril of relapse (Han et al., 2022). Chemotherapies like platinum-based drugs (carboplatin and cisplatin) have been reported to cause nephrotoxicity, cardiovascular diseases, and low-grade gliomas in up to 47% of children. Lavniti M. et al. have reported that oxaliplatin and etoposides have also induced neuropathy, which may cause permanent gastrointestinal dysfunctions with CNS depression in cancer-afflicted and treated patients (Eiermann et al., 2001; Shipitsin and Polyak, 2008). Tachycardia, decreased immunity, and bone marrow depression are major drawbacks of antibody-based treatment (rituximab) and targeted therapies. Surgery and radiation therapy for cancer management induce anorexia, lymphedema, neutropenia, sepsis, hair-loss, and immunological disorders (Spychalski et al., 2007). Many of the adverse effects of cancer treatments are managed by some phytoconstituents. However, treatments are always associated with various types of adversities. Hence, scientists and clinicians around the globe advocate the use of dietary phytoconstituents to prevent cancer progression. Interestingly, the amount of phytoconstituents present in diets is not clinically significant enough to block the cancer progression if initiated (Liu et al., 2019). Plant-derived phytomolecules (capsaicin, resveratrol, fisetin, 6-gingerol, etc.) have been utilized in the treatment of cancer and have provided evidence for their intervention in different cancer progression pathways, as discussed below. Recently, the focus has shifted toward cancer prevention with phytochemicals obtained from natural sources. These phytochemicals can target effector molecules in cancer-developing pathways and are the major focus of this manuscript.

## 2 Signaling pathways and molecules as targets for the prevention of cancer

Signaling pathways are chains of chemical processes where several molecules in a cell interact to regulate a cell function, such as cell division or cell death. Targeted medicine in cancer treatment utilizes molecular modifications in cancer genes and signaling pathways to influence the development of new therapies. Different pathways of cancer progression, like JAK-STAT, Wnt-β-catenin, NF-κB, MAPK pathways, and different signaling molecules, have the tendency to crosstalk with other signaling pathways. These signaling pathways that serve as potential targets in cancer prevention have been discussed in the subsequent sub-sections.

### 2.1 JAK-STAT pathway

**FIGURE 3:**
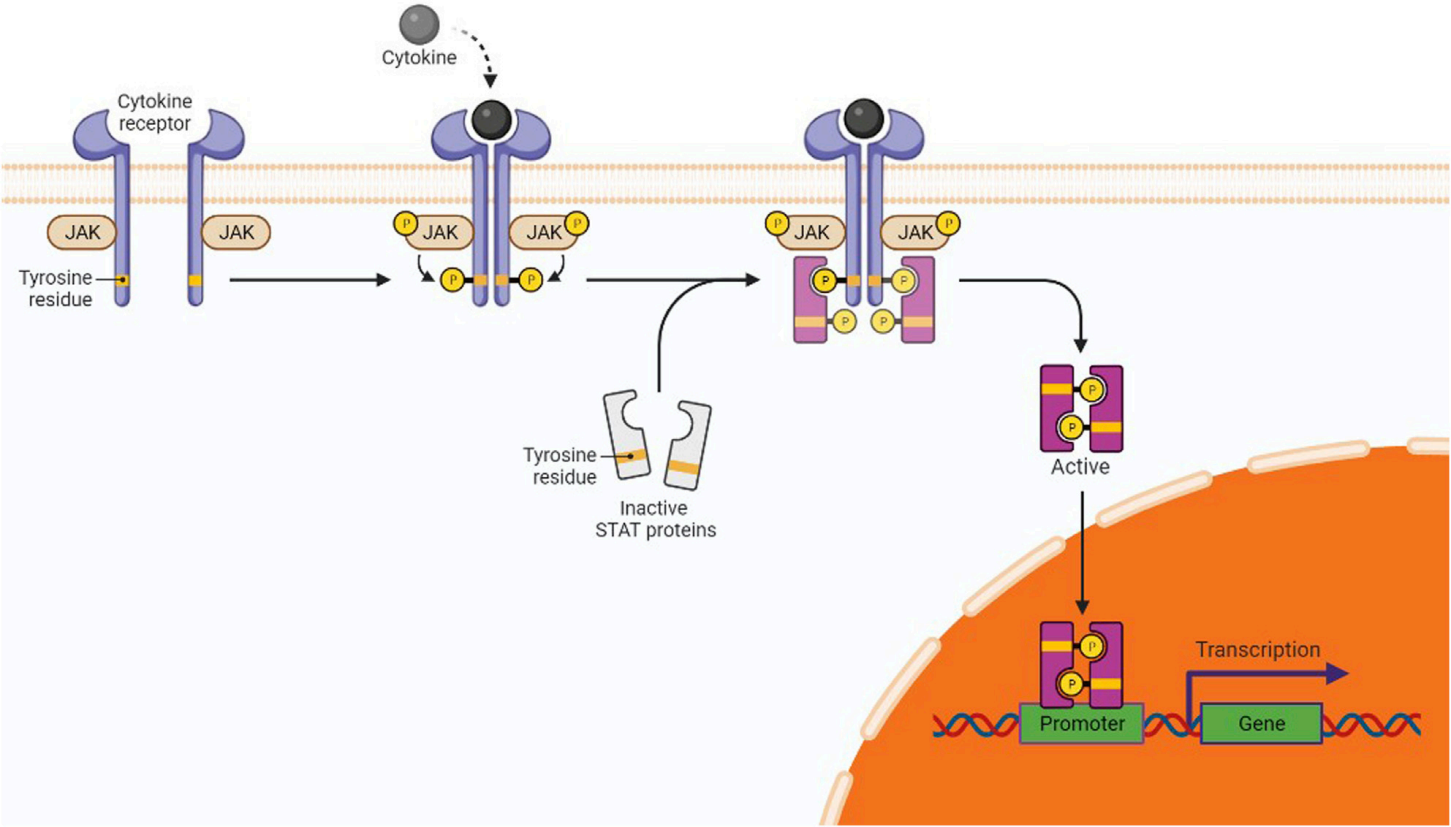
JAK/STAT in gene expression and cancer pathway. The interaction of cytokines or growth factors with their receptors (cytoplasmic tails of membrane cytokine receptors) induces dimerization/oligomerization of these receptors by inducing a conformational change in the cytoplasmic domain. JAKs or other families of tyrosine kinases are auto-phosphorylated or trans-phosphorylated as a result of this interaction. The phosphorylated JAKs form the sites for the binding of other signaling molecules with an SH2 domain (like STAT proteins) by phosphorylating the cytoplasmic tails of the receptor on tyrosine residues. Cytoplasmic STATs bind to phosphorylated receptors. STATs can assemble into homodimers or heterodimers that can translocate to the nucleus and activate gene transcription.

About 50% of breast cancer and leukemia and 90% of cervical cancer are caused by dysregulations in the Janus kinase (JAK)/signal transducer and activator of transcription (STAT) transduction pathway (JAK/STAT pathway) (Hu et al., 2013). This pathway serves as a crucial mediator in many cellular processes, including cell death, differentiation, and apoptosis (Shao et al., 2020). When cytokine-mediated glycoproteins like interferons, interleukins, and growth factors interact with cytokine receptors (CRs) on the surface of the target cell, it activates JAK molecules through transphosphorylation in endothelial and vascular smooth muscles (Loscocco and Vannucchi, 2022). Activated JAK initiates the phosphorylation of tyrosine residues located in the cytoplasmic region of CRs, which creates the binding positions for STATs (Hu et al., 2021). STATs are phosphorylated on tyrosine residues by tyrosine kinase, leading to the formation of STAT homo/hetero dimers in the cytosol. These dimers further translocate to the nucleus, bind with deoxyribose nucleic acid (DNA), and regulate the transcription of myc, p21, and Bcl-xl (Figure 3) (Bose et al., 2020). Dimers of unphosphorylated STATs can also localize to the nucleus and bind to heterochromatin protein 1. This further promotes heterochromatin formation, resulting in transcriptional upregulation. Typically, STATs are supposed to initiate the transcription of genes, but their unphosphorylated form can also downregulate the expression of several genes by interacting with HP1α and acting as a tumor suppressor (Brooks and Putoczki, 2020).

Escalated expression of negative regulators of the JAK/STAT pathway, *viz.*, the suppressors of cytokine signaling (SOCS), can reduce pathway activation and tumorigenesis as well. Expression of SOCS/SOCS3 reduces the constitutive phosphorylation of STAT molecules at the tyrosine residues of CRs. Brantley et al. have reported the cessation of oncogenesis and transcription of genes due to SOCS expression in different tumor samples as it downregulates the secretion of cytokines; hence, the JAK-STAT pathway is not activated (He et al., 2003).

### 2.2 Wnt β-catenin pathway

**FIGURE 4:**
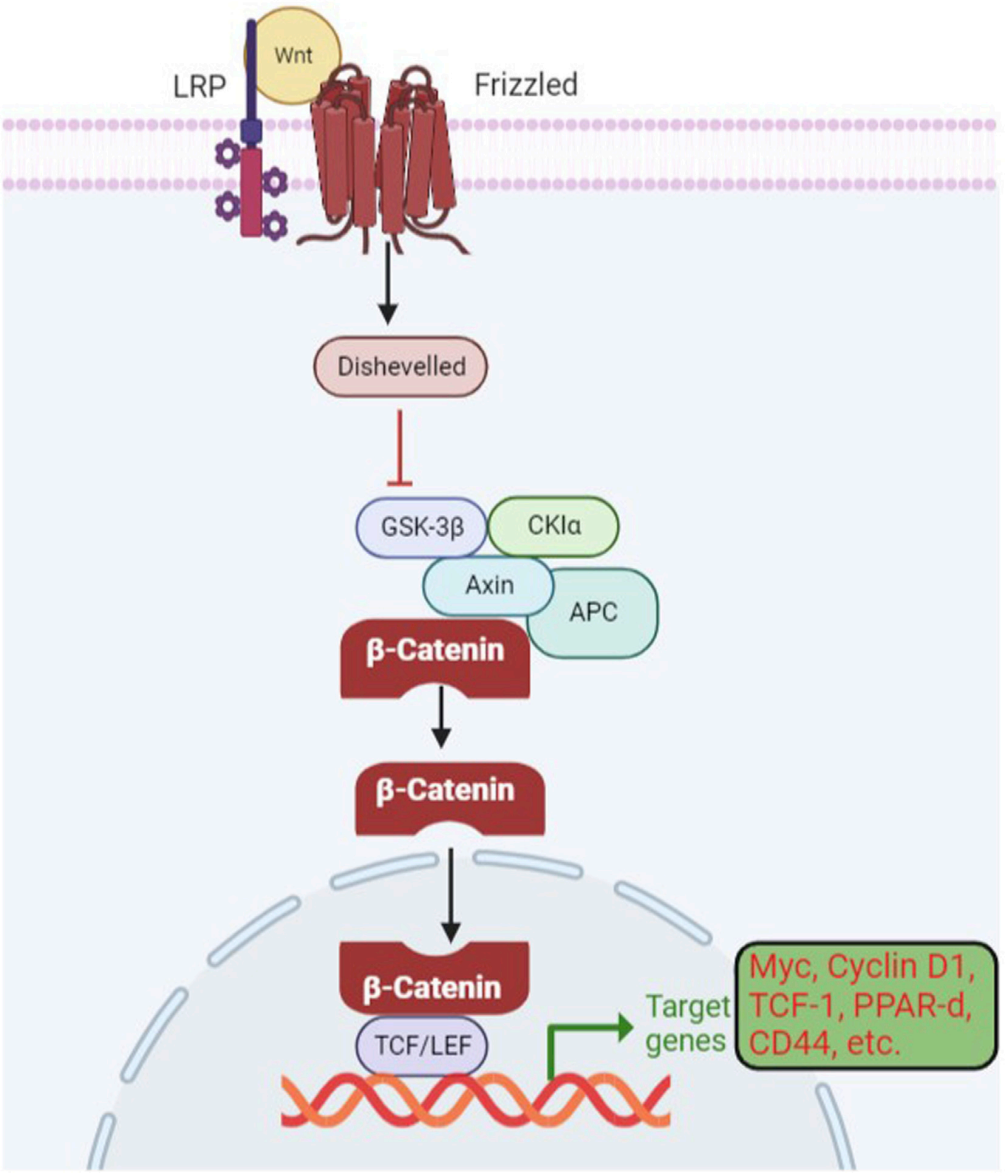
Wnt β-catenin signaling pathway in gene expression and cancer development. When Wnts are attached to their LRP5/6 and frizzled protein receptors, the cytoplasmic protein DVL is activated, which results in the inhibition of GSK3 in the destruction complex. The target gene is then transcriptionally regulated as a result of stabilized catenin binding to the TCF/LEF transcription factors in the nucleus.

Wnt-β signaling has been found to be active in over 50% of breast cancer and 25% of melanoma. Dysregulation of the Wnt β-catenin pathway has been reported in various types of cancer, including breast, lung, pancreatic, ovarian, colorectal, and gastric cancer (Zhan et al., 2017). Low-density lipoprotein receptor-related proteins (LRP) and frizzled receptors form a complex as a result of the interaction with a wingless-related integration site (Wnt) (Deitrick and Pruitt, 2016). The frizzled receptor recruits disheveled protein (DVI), which leads to the phosphorylation of the LRP receptors. It causes the inactivation of the destruction complex composed of Axin, adenomatosis polyposis coli (APC), casein kinase (CK), and glycogen synthase kinase (GSK). It disrupts the Axin-mediated degradation of β-catenin, as shown in Figure 4. The translocation and accumulation of β-catenin in the nucleus causes the replacement of Groucho in the nucleus and leads to the activation of the Wnt-responsive genes (Krishnamurthy and Kurzrock, 2018; Shen et al., 2022). Erythroblast transformation-specific (ETS)-related genes (ERGs) have also been reported to control the Wnt-signaling pathway by stabilizing β-catenin. In mouse models, ERG has been shown to regulate the secretion of vascular endothelial growth factor (VEGF) and vascular stability through Wnt signaling (Daisy Precilla et al., 2022). It is possible because ERG has been reported to have the potential for the transcription of frizzled class 4 receptors and simultaneously provide stability of β-catenin in endothelial cells (ECs). A reduced level of β-catenin has been associated with reduced tumor formation (Patel et al., 2019; Liu et al., 2021).

### 2.3 p53 gene: association with cancer progression

**FIGURE 5:**
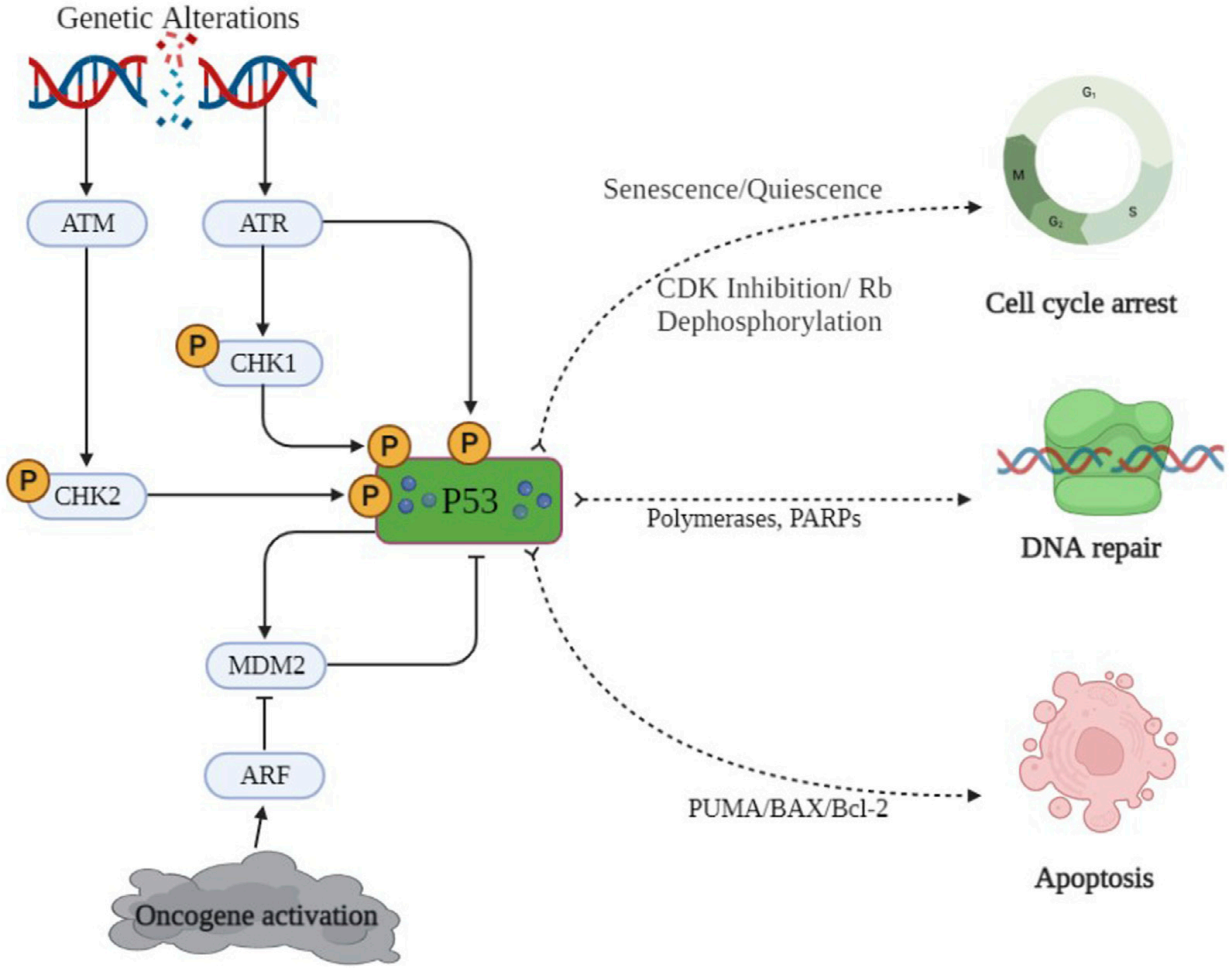
p53 signaling pathway. DNA damage and oncogene activation is only a few of the cellular stressors that activate the sensor proteins ATM and CHK2. Murine double-minute 2 (MDM2), which is a target of p53 and also controls p53 stability, creates a negative feedback loop. Activated p53 is involved in numerous pathways, including apoptosis pathways, cell proliferation, and DNA repair and cell cycle arrest.

The p53 protein plays a pivotal role in the transcription of several genes and also acts as a tumor suppressor. It is often referred to as the “guardian of the genome” because of its ability to detect DNA damage and promote either DNA repair or programmed cell death in damaged cells. Over 50% of human cancer involves the deactivation of p53 or mutations in p53 (Li, 2021). Whenever oxidative stress or genetic alterations initiate DNA damage, an automated correction mechanism gets activated via the protein sensor of DNA damage, i.e., ATM/CHK2. This, in turn, phosphorylates p53, which may check the damage through either of the transducer mechanisms, as shown in Figure 5 (Nakano and Vousden, 2001). p53 has also been reported to trigger PUMA and BAX molecules to accelerate the apoptotic pathways. p53 is involved in the inhibition of the cyclin-dependent kinases (CDK) complex, causing Rb dephosphorylation, resulting in cell cycle arrest (Akhter et al., 2014). Qiu L et al. reported that p53/p21 disbalance affects many biological processes, including cell proliferation by arresting the cell cycle at the G2/M phase and apoptosis. p53 has been reported to inhibit epithelial–mesenchymal transition (EMT) (Borsos et al., 2022) by reducing the levels of zinc finger E-box-binding homeobox 1 and 2 (ZEB1 and ZEB2) expressions (Banin et al., 1998). To sum up, p53 may either activate DNA repair enzymes (PARPs and polymerases), cause temporary cell cycle arrest at the G1 phase to remove the damaged portions (quiescence/senescence), or accelerate the secretion of PUMA/BAX to induce apoptosis in cancerous cells, as depicted in Figure 5. So, the activation of p53 through small molecules might be a key to preventing damaged cells’ conversion into cancerous cells (Yu et al., 2001).

### 2.4 Cyclin D and CDK complex

Cyclin D is a member of the cyclin protein family and is involved in the regulation of the cell cycle and its functions. The cyclin D/cyclin dependent kinase-4 (CDK-4) complex is a multi-protein structure made up of cyclin D and CDK4, a serine–threonine kinase (Bury et al., 2021). The synthesis of cyclin D starts with the action of growth factors. Cyclin D_1_ protein converts the inactivated form of CDK4/6 to its activated form and mediates the G1/S phase transition (Zhang et al., 2021; Liu et al., 2022). This further triggers the phosphorylation of the Rb protein and leads to uncontrolled cell proliferation. Overexpression of cyclin D1 has been observed in a wide range of cancers, including breast cancer, lung cancer, and prostate cancer (Li et al., 2022). It is associated with poor prognosis and resistance to cancer treatment. Cancer preventive agents have been reported to inhibit the cyclin D1 protein and its complex, which further decreases cell proliferation by dysregulating cyc-D expression and results in cell cycle arrest. CDK/cyc-D inhibitors have been developed to control cancer progression and are currently under clinical trials, like abemaciclib and ribociclib (Heptinstall et al., 2018).

### 2.5 TNF-α/NF-κB signaling pathway

**FIGURE 6:**
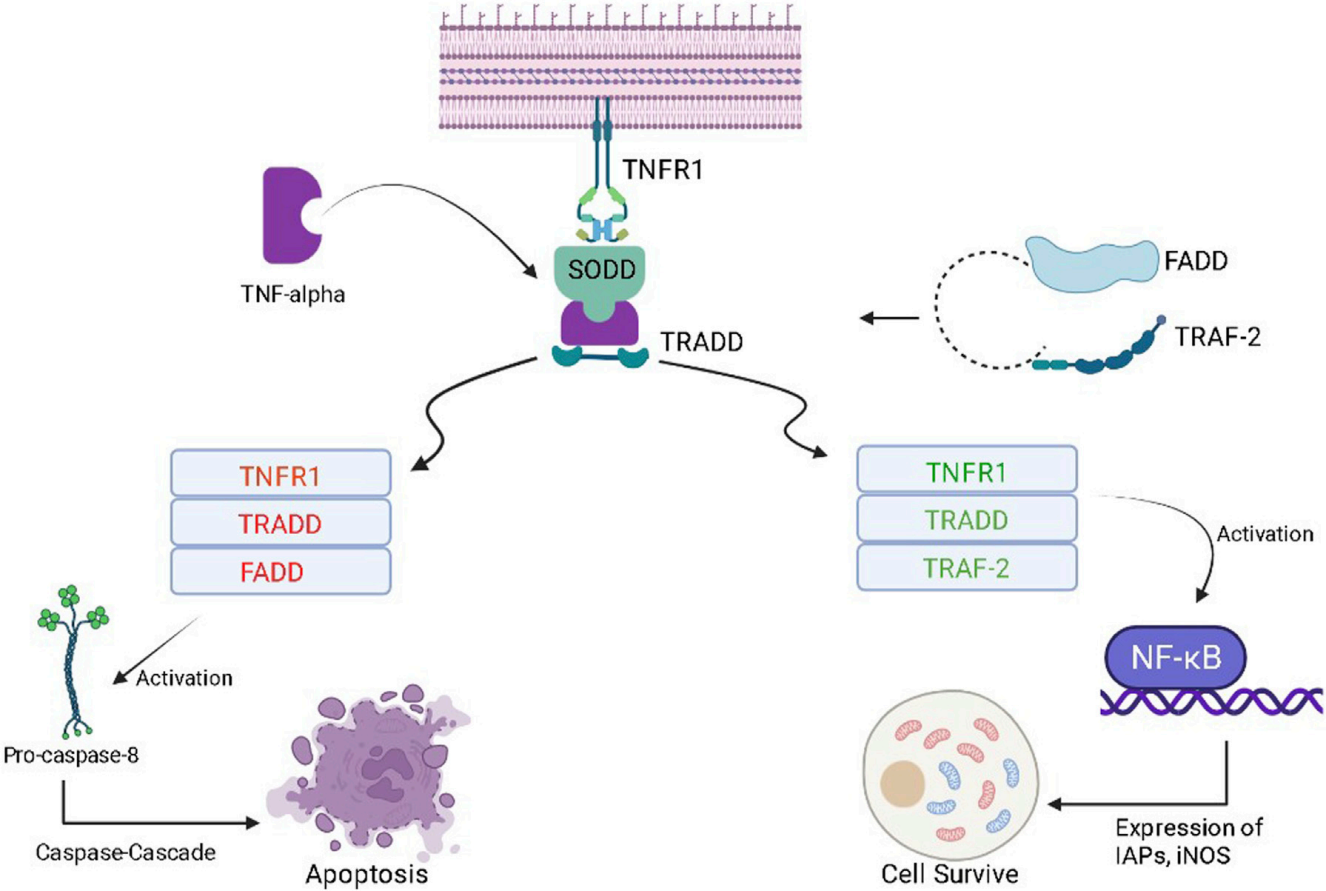
Role of the TNF-α in the progression of cancer and programmed cell death.

Tumor necrosis factor-α (TNF-α) is a cytokine that plays a complex role (pro-inflammatory and anti-tumor) in cancer patients. In the early stages of cancer development, TNF-α can stimulate the immune system, attack cancer cells, and lead to apoptosis of tumor cells. However, in the later stages of cancer development, chronic inflammation can occur, which can promote the growth and spread of cancer cells. TNF-α can also stimulate the production of angiogenic factors, which can act as fuel for the growth of tumors. At the molecular level, when TNF-α binds to its receptor present at the cell surface of a normal healthy cell, it gets inactivated due to the presence of the silencer of the death domain (SODD) (Alharbi et al., 2021). The binding of TNF-α with SODD restores the activities of the tumor necrosis factor receptor (TNFR), and SODD is liberated/released. This complex is attacked by TNF-R1-associated death domain protein (TRADD) and turns into the TNFR-TRADD complex (Aggarwal et al., 2006). Further transduction depends on the type of protein molecule that gets attached to the complex. If FAS-associated death domain (FADD) molecules bind with this complex, it causes the release of pro-caspase 8, which further causes apoptosis of cancerous cells using caspase-cascade. On the other hand, if TNF receptor-associated factor (TRAF-2) interacts with the TNFR-TRADD complex, it causes the activation of the inhibitor of apoptotic proteins (IAPs) and, hence, nuclear factor- κB (NF-κB) is activated (Figure 6) (Liu W. et al., 2020). NF-κB further promotes cell survival by increasing the expression of the cellular inhibitor of apoptosis protein-1 (cIAPs) and inducible nitric oxide synthase (iNOS). Whenever there is a reduction in the activity of cytoplasmic IAPs and receptor-interacting protein (RIP1) kinase, receptor internalization occurs. This further results in the activation of caspase 8 and, then, causes the apoptosis of the particular cell (Aggarwal et al., 2002). Whenever there is an increase in cytoplasmic IAPs, it retards the activity of the electron transport chain in the mitochondria. This further activates reactive oxygen species (ROS), then RIP-1 is recruited, and the process of NF-κB activation starts again (Storci et al., 2010). In cancerous cells, after the formation of the TRADD-SODD complex, it has a higher affinity for TRAF-2 than the FADD molecule. This causes increased NF-κB production inside cancerous cells and leads to cell survival by preventing apoptosis. The manipulation of TNF-α and its downstream pathways may offer newer therapeutic opportunities for cancer prevention.

### 2.6 Regulation of apoptotic molecules

**FIGURE 7:**
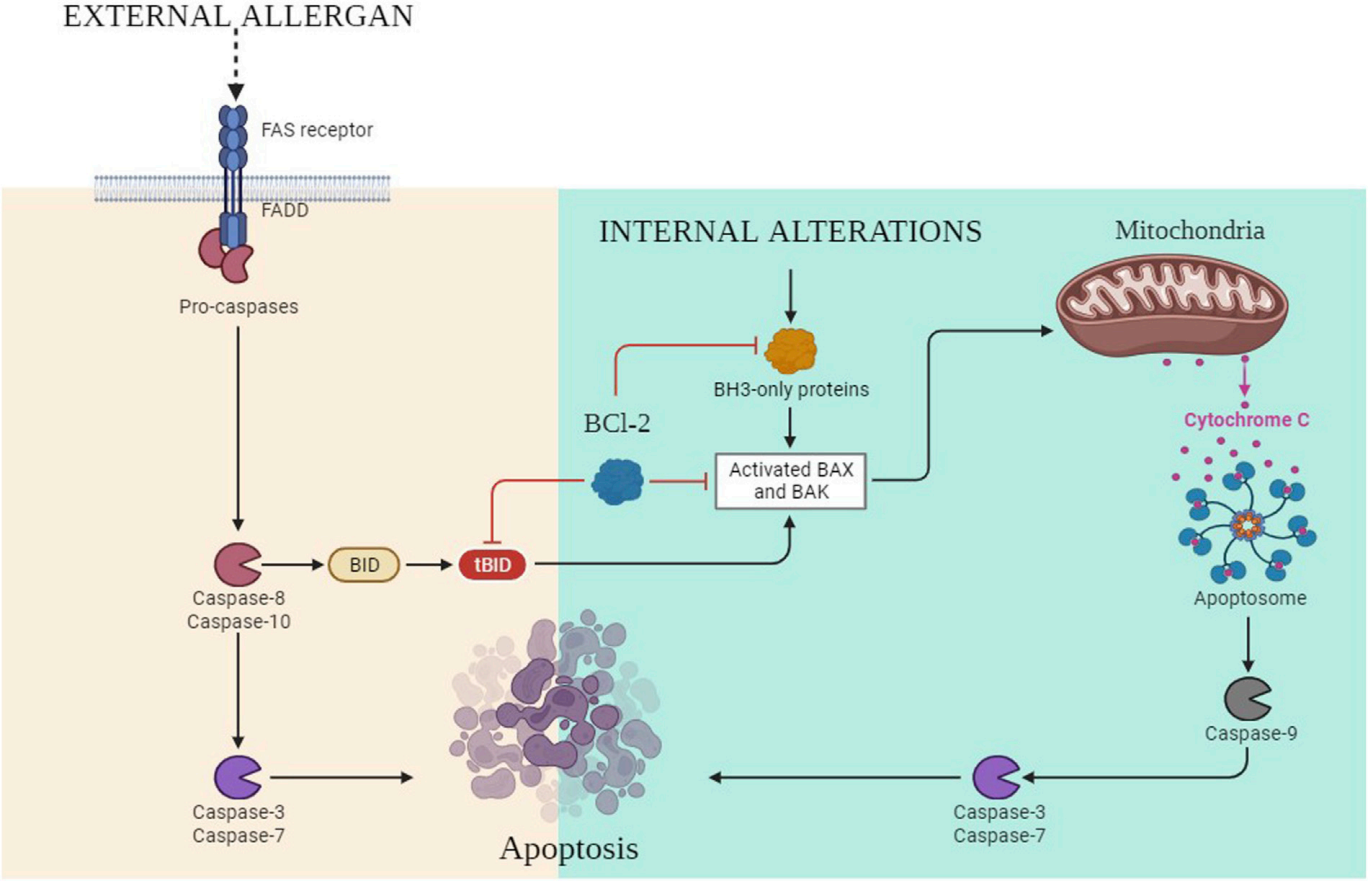
Intrinsic and extrinsic pathways of apoptosis.

Programmed cell death or apoptosis of any cell relies on three steps, i.e., initiation, execution, and phagocytosis. It is an essential biological process that plays a critical role in various physiological conditions, including cancer. In the intrinsic pathway of apoptosis, DNA lesions release ataxia-telangiectasia mutated (ATM) serine and threonine kinase (Plati et al., 2011). These activate p53, which further upregulates the p53-upregulated modulator of apoptosis (PUMA) (Hassan et al., 2014; Goldar et al., 2015). B-cell lymphoma-2 (Bcl-2)-associated X protein (BAX) is activated in the cytosol of the cell by the upregulation of PUMA, and it opens the voltage-dependent anion-selective channel (VDAC) on the surface of the mitochondria. It results in the release of cytochrome C out of the cytosol (Duan et al., 2023). It binds with pro-apoptotic apoptosis protease-activating factor 1 (APAF-1), and the apoptosome is activated (Verbrugge et al., 2010; International, 2020), which further activates caspase 9 and caspase 3 (Figure 7). Furthermore, the activation of nuclease and protease causes DNA fragmentation, which leads to cell death. In the extrinsic pathway, the signaling is attributed to TNF-α and FS-7-associated surface antigen (FAS) molecules. These molecules activate TNF or FAS receptors. The death-inducing signaling complex (DISC) complex is formed (Ouyang et al., 2012). The interaction of FAS with the FAS receptor causes the activation of FADD, which further releases pro-caspase 8 and caspase 3. This caspase 3 activates caspase-dependent DNAase and proteolytic enzymes, which in turn degrade DNA and proteins, culminating in cell death (Clarke and Tyler, 2009). B-cell lymphoma-2 (Bcl-2) is the negative regulator of this pathway, which has antagonistic actions for BAX. In the extrinsic pathway, the conversion of the BH3 interacting domain (Bid) to truncated (t-Bid) due to the activation of the caspase-cascade complex releases BAX in the cytosol, which might then start the further process of the intrinsic pathway (Xu Y. et al., 2020; Yang et al., 2020). In cancerous cells, due to the dysregulation of p53, PUMA has not been activated. This further leads to decreased BAX production; hence, as a result of this, the caspase-cascade system does not get activated, and ultimately, the cells survive.

### 2.7 Association of VEGF with metastasis

**FIGURE 8:**
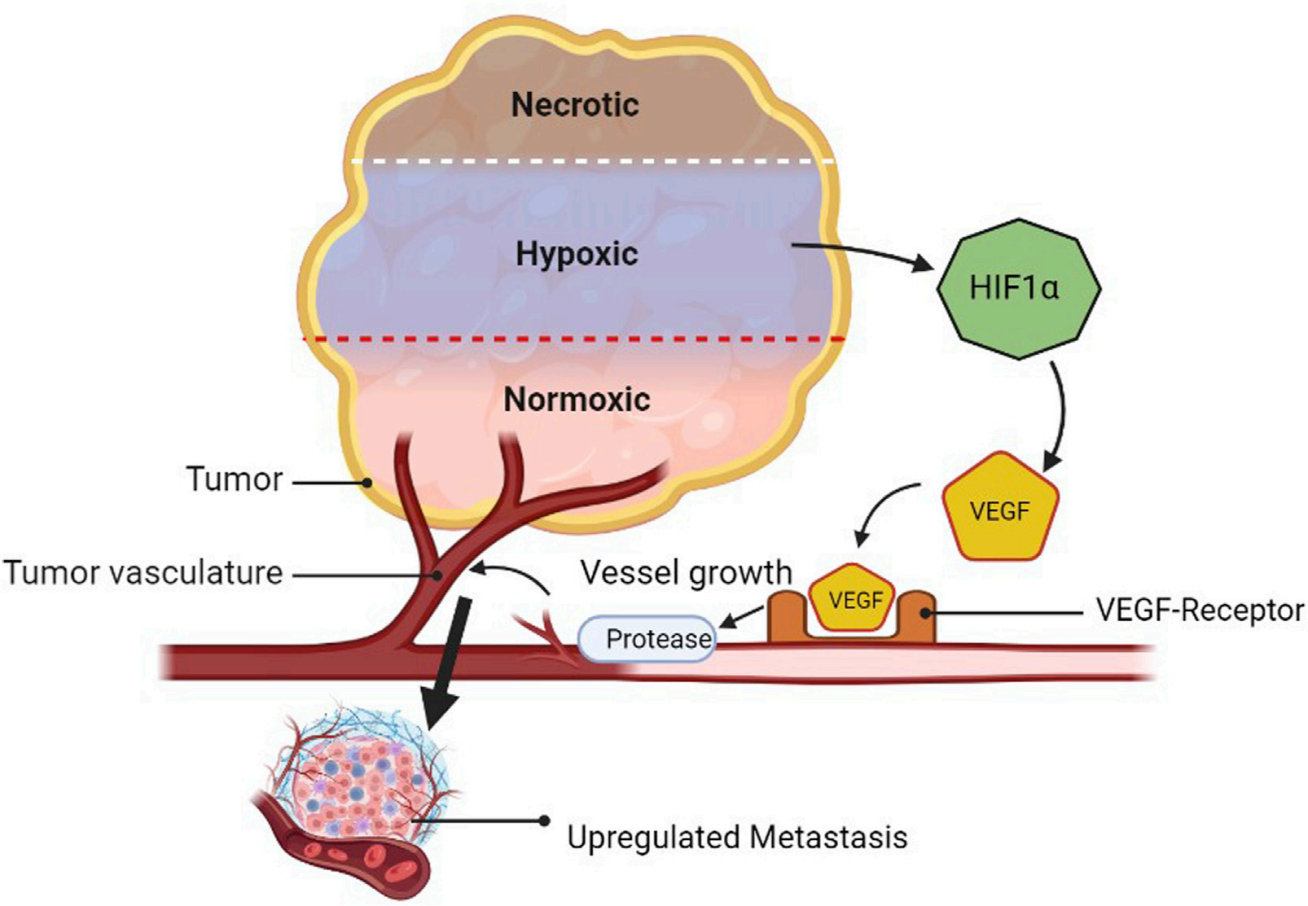
Amplification of metastasis through VEGF upregulation.

Angiogenesis is the process by which new blood vessels are formed from pre-existing vessels. Tumor microenvironment features include cell recruitment, release of soluble factors, and hypoxia, all of which contribute to angiogenesis. Angiogenesis is also characterized by resistance to anti-angiogenic therapy. The development of cancerous cells requires a high amount of oxygen and nutrients and releases HIF1α in comparison to healthy cells. Furthermore, it releases the VEGF. The interaction of the VEGF with its receptor (VEGF-R) secretes proteases that make the blood vessels leaky and lead to the generation of new blood vessels toward growing cancerous cells. When the platelets of normal or cancerous cells are drawn to hypoxic areas, they become activated and release the stimulatory substances they have stored there into the tumor microenvironment. The bone marrow’s endothelial progenitor cells (EPCs) and myeloid cells relocate to the tumor microenvironment, where they locally release more soluble factors (Nishida et al., 2006; Lv et al., 2023). The tumor cells become more invasive in this environment and are able to metastasize to distant organs by intravasation into the lymphatics or vasculature (Figure 8) (Rajabi and Mousa, 2017). Valid regimens for cancer prevention must consider the targets of multiple cell types that are targetable for angiogenic factors, such as platelet-derived growth factor (PDGF), PDGF receptor (PDGFR), angiopoietin 1/2 (Ang1/2), ECs, and EPCs (Rajabi and Mousa, 2017).

## 3 Role of plant-derived secondary metabolites in cancer prevention

Phytochemicals, including paclitaxel, docetaxel, and resveratrol, have shown anticancer activity and completed clinical trials (Choudhari et al., 2020). Phyto-analogs have shown desirable effects on p53, BAX, VEGF, β-catenin, BAK, Bcl-2, CDKs, and interleukins. Some secondary metabolites work either by preventing the formation of cancer cells or by inducing the death of existing cancer cells. For example, flavonoids, a class of secondary metabolites found in many fruits and vegetables, have been shown to inhibit the growth and proliferation of cancer cells *in vitro* and *in vivo*. Polyphenols, another class of secondary metabolites found in many plant-based foods, have been shown to have similar anticancer properties (Hosseini and Ghorbani, 2015). Overall, secondary metabolites play an important role in cancer prevention by providing a natural source of anticancer compounds that can be incorporated into a healthy diet. Although more research is needed to fully understand the mechanisms underlying their anticancer properties, consuming a variety of fruits and vegetables rich in secondary metabolites is likely to have important health benefits, including reducing the risk of cancer. Here, in this section, we have divided selected herbal plants into their classes as per their metabolic nature, and the activity of these drugs has been summarized in Table 1.

**TABLE 1:** Summarized reviews of the botanicals used in cancer prevention.

Cancer-preventive phytomolecule	Botanical source	Metabolite class	Dose	Molecular target and mechanism of action	Cancer category	Animal/cell lines	Reference
Curcumin	Rhizomes of *Curcuma longa*	Flavonoid	20 µM	Inhibits NF-kB activation, upregulates SOCS expression, inhibits JNK activation, and phosphorylates Bcl-2, leading to autophagic cell death in cancerous cells	Breast cancer	MCF-7 and MDA-MB-231	Wang et al. (2016)
Silymarin	Fruits of *Silybum marianum L.*		200 mg/kg, topically, two weeks	Inhibits TNF-a and NF-kB activation and targets BAX/BAK and BCl-2 levels	Squamous cell carcinoma	Old SENCAR mice	Wang et al. (2023)
Ginestin	Seeds of *Glycine soja sieb.*		500 microgm/kg body weight on three alternative days	Inhibits cyclin D kinases, cell proliferation, angiogenesis, invasion, metastasis, and apoptotic processes targeting BAX/Bcl-2 levels	Breast cancer	C57Bl/6 mice	Su et al. (2005)
Fisetin	Fruits of *Fragaria virginiana*		100 µM	Inhibits the Wnt signaling pathway and proliferation of cancerous cells	Nasopharyngeal carcinoma	CNE1 and CNE1-LMP1 cell lines	Suh et al. (2009)
Baicalin	Roots of *Scutellaria baicalensis*		15 mg/kg/day, i.p. route, two weeks	Inhibits NF-kB/IL-1β, VEGF amplification, and Bcl-2 and upregulates apoptotic caspase-3, pro-apoptotic p53, and Bax	Breast cancer	Female Swiss albino mice	Shehatta et al. (2022)
Apigenin	Flowering tops of *Petroselinum crispum*		5 µM	Active against VEGF and reduces tumor volume and cell proliferation	Skin cancer	NHEK cell lines	Li et al. (2020a)
Lycopene	Fruits of *Solanum lycopersicum*	Terpenoid	5 µM	Inhibits VEGF, BAX, NF-kB, MEK, and CDKs and upregulates p53 levels, leading to chemoprevention and anti-angiogenic potential	Colorectal carcinoma	HCT116, SW480	Xu et al. (2020a)
Dihydrotanshinone	Roots of *Salvia miltiorrhiza*		5 µM	Downregulates PUMA, BAX, and EGFR in apoptotic pathways and boosts p53 and Bcl-2 levels	Liver cancer	HepG2 and HT-29 cells	Jiang et al. (2019)
β-carotene	Fruits of *Daucus carota*		15 µM	Accelerates BAX levels and inhibits Bcl2, NF-kB, and NRF-2, promoting cancer-prevention activity	Colon cancer	HCT116, SW480	Kavalappa et al. (2019)
Artemisinin	Shrubs of Artemisia annua		300 mg/kg	Membranous translocation of beta-catenin and inhibition of unrestricted activation of Wnt/b-catenin pathway, leading to cancer-prevention activity	Breast cancer	Balb/c female, nude mice	Li et al. (2007)
Eugenol	Flowering tops of *Syzygium aromaticum*		150 µM	Inhibits Nf-kB activation and target IL-1B levels	Breast cancer	MCF-2 cells	Sarkar et al. (2020)
Anethole	Dried and ripe fruits of *Foeniculum vulgare*		8 mg/kg, oral, one week	Inhibits cell proliferation and NF-kB activation pathway	Breast cancer	Female Sprague–Dawley rats w	Lubet et al. (1997)
Capsaicin	Dried fruits of *Capsicum annum*	Alkaloid	50 µM	Inhibits STAT and NF-kB activation and modulates SOCS and IkB α levels	Gastric cancer	AGS cells	Gilardini Montani et al. (2015)
Sanguinarine	Rhizomes of *Sanguinaria canadensis*		10 µM	Inhibits proliferation and NF-kB activation pathway. Increases apoptosis and necrosis in cancerous cells	Epidermoid carcinoma	A431 and NHEK cells	Achkar et al. (2017)
6-Gingerol	Rhizomes of *Zingiber officinale*		100 mg/kg, oral, 120 days	Inhibits cell proliferation by targeting TNF-α, IL-1β, iNOS, COX-2, and cyclin D1 and upregulates APC and p53, yielding cancer-preventive activity	Colorectal cancer	Male BALB/c mice	Farombi et al. (2020)
EGCG	Fresh leaves of *Camellia sinensis*		100 µM	Inhibits NF-kB activation, VEGF, BAX, NF-kB, MEK, and CDKs and modulates SOCS and Bcl-2 levels	Oral cancer	HSC-3 cell lines	Wang et al. (2018); Wei et al. (2022)
Resveratrol	Fruits of *Vitis vinifera*		150 µM	Inhibits NF-kB and JAK-STAT activation pathways and upregulates SOCS and Bcl-2 levels, activating apoptosis in cancerous cells	Squamous cell carcinoma	SCCHN cells	Baek et al. (2016)
Caffeic acid phenethyl ester (CAPE)	Dried and ripe fruits of *Coffea arabica*		20 mg/kg, oral, 10 days	CAPE has been reported to act against the NF-kB activation pathway and gene expressions	Liver cancer	Male Wistar rats	Carrasco-Legleu et al. (2004)
Allicin	Fruit bulbs of *Allium sativum*	Organometallic compound	100 mg/kg, oral, 5 times a week	Inhibits iNOS, NF-kB activation, and VEGF, showing cancer-prevention activity	Colorectal cancer	A/J mice	Su et al. (2021b)
Sulforaphane	Fruits of *Brassica oleracea*		15 µM	Decreases NF-kB and tumor size and inhibits CDK	Breast cancer	SUM149 and SUM159	Burnett et al. (2017)
Vitamin D3	Cheese or fish liver oil		200 µM	Inhibits p-Bcl-2, c-Myc, p-IGF-IR, p-mTOR, p-P70S6K, and p-S6 and upregulates caspase-3, BAX, and p-AMPK levels	Amelanotic melanoma	Hs695T	Nica-Badea and Udristioiu (2021)
Extract of *Syzygium samarangense*	Seeds of *Syzygium samarangense* (SS)	-	500 µg/ml	Inhibits c-Myc and NF-kB activation and upregulates apoptosis in cancerous cells by interacting BAX/Bcl-2 levels	Liver cancer	HEP G2-C8 cells	Su et al. (2021b)

### 3.1 Role of flavonoids

Plant species contain a group of secondary polyphenolic compounds known as flavonoids, which are generally included in human diets. Chemically, flavonoids have a 15-carbon skeleton that is made up of two phenyl rings and a heterocyclic ring. The abbreviation for this carbon structure is C6–C3–C6. Flavonoids are known for diverse pharmacological activities (Nagoor Meeran et al., 2021; Singh et al., 2023).

Apigenin is classified as a flavone among the different classes of flavonoids. It is abundantly present in common fruits such as grapefruit, plant-derived beverages, and vegetables such as parsley, onions, oranges, tea, chamomile, wheat, sprouts, and some seasonings (Li Y. et al., 2020). The chemo-preventive role of this flavonoid (apigenin) has been tested on wild-type and specially modified mice (female Swiss albino mice) (Ganjare et al., 2011; Hang et al., 2022). It has shown a reduction in angiogenesis by reducing the VEGF level in breast cancer (Ai et al., 2017; Meng et al., 2017). The effect of apigenin was recorded in UVB-induced cancers in wild-type (WT) and modified (TKO) mice. Apigenin has shown significant cancer prevention and recovery by reducing tumor volume and VEGF compared to the negative control. Apigenin has also shown a reduction in blood capillary formation in the apigenin-treated mice group compared to that in other groups (Ai et al., 2017; Meng et al., 2017).

One such secondary metabolite, or flavonoid, is curcumin. Curcumin is a non-toxic diarylheptanoid belonging to the group of curcuminoids. It is obtained from the dried rhizome of *Curcuma longa* Linn. belonging to the family Zingiberaceae (Oliveira et al., 2021). Curcumin has been reported for its cancer-preventive effects on different breast cancer cell lines (MCF-7 and MDA-MB-231 cell lines). It has been reported to boost autophagy in cancerous cells. It has shown stimulatory effects on apoptotic enzymes (BAK/BAX). It has also been reported to boost p53 levels in MCF-7 cell lines (Wang et al., 2016). Curcumin has also shown synergistic actions with berberine and potentially alters the levels of STATs, Β-catenin, Bcl2, BAX, p53, NF-κB, and CDKs. In WIPO PCT, a patent has been filed for the cancer-preventive effects of curcumin and turmeric oil with the application number WO2014068597A2, in which the drug has decreased the levels of NF-κB and reduced the proliferation of cancerous cells.

Another flavonoid involved in cancer-preventing activities is silymarin. Silymarin is a standardized milk thistle seed extract containing a mixture of flavonolignans (*Silybum marianum*) belonging to the group of flavonoids (Abouzid et al., 2016). Silymarin has been reported to inhibit organic anion transporters (OAT) and ATP-binding cassettes (ABC) transporters, showing cancer-preventive effects. It has also been reported to boost the activity of apoptotic molecules and BAX, which activates the caspase system of apoptosis in 6 -week-old SINCAR mice with squamous cell carcinoma (Malekpour-Dehkordi et al., 2022). It has also reduced tumor volume and prevented tumorigenicity by reducing TNF-a levels (Wang et al., 2023).

Ginestin is a phytoestrogen belonging to the flavonoid group. It is found in soybeans and soybean-enriched products (Cools et al., 2014). At the molecular level, ginestin is active against CDKs, cell proliferation, angiogenesis, invasion, metastasis, and apoptotic processes. Ginestin has shown effects on Akt, NF-κB, matrix metalloproteinases (MMPs), and BAX/Bcl-2 signaling pathways for breast cancer prevention in C57Bl/6 mice (Sarkar and Li, 2002). It has also been reported to show synergistic effects with resveratrol via boosting the expression of p53 and diminishing the NF-kB pathway of cancer development (Spagnuolo et al., 2015).

One more flavonoid reported to have cancer-preventive potential is fisetin. Fisetin is a flavanol from the flavonoid group of polyphenols. It is obtained from fruits and vegetables such as strawberries, apples, persimmon, grapes, onions, and cucumber (Gleńsk et al., 2021). Fisetin is also a good antioxidant and is involved in the inhibition of the Wnt signaling pathway. It has been reported to inhibit YB-1 protein and proliferation in nasopharyngeal carcinoma. This study was performed on CNE11 and CNE1-LMP1 cell lines of nasopharyngeal carcinoma (Ravula et al., 2021).

One more glycosyloxyflavone flavonoid with cancer-preventing potential is baicalin. Scutellaria baicalensis (SB) extract contains baicalin, which is a natural antioxidant, belonging to the family *Lamiaceae* (Li et al., 2004). Baicalin has been reported to prevent tumor formation by increasing the levels of p53 and BAX against breast cancer induced in Swiss albino mice (Zhao et al., 2016; Huang et al., 2022). Baicalin has also been reported to reduce the levels of ILs, Bcl2, and VEGF, showing cancer-preventive effects (Shehatta et al., 2022). Baicalin has shown its synergistic effects along with 5-fluorouracil (5-FU).

### 3.2 Role of terpenoids

Isoprene is utilized to make a group of chemical compounds known as terpenoids, commonly known as isoprenoids. Terpenes and the 55-carbon molecule isoprene are two examples of organic compounds that are found in nature. These are multi-cyclic structures with oxygen-containing functional groups. Examples include lycopene, dihydrotanshinone, and carotene. Lycopene, a naturally occurring carotenoid, is extracted from tomatoes, belonging to the Solanaceae family (Adetunji et al., 2021). Lycopene has shown a chemo-preventive effect by inhibiting the NF-κB pathway and Bcl2, which further stops cancer proliferation in HCT116 and SW480 cell lines of colorectal carcinoma (Adetunji et al., 2021). It has also reduced lung and liver inflammation by activating SIRTUINS and has been shown to alter the levels of oncogenes like NP73 and upregulate p53 to prevent proliferation, as evaluated against colorectal cancer cell lines (Lim and Wang, 2020). Lycopene has also shown synergistic effects in combination with β-cryptoxanthin. According to the IPO, no patent has been filed for its chemo-preventive efficacy; however, its efficacy has been reported for protecting the skin from UV radiation.

Dihydrotanshinone is another example in this class. Dihydrotanshinone belongs to a class of lipophilic diterpenoids and is extracted from *Salvia miltiorrhiza* Bunge (Wang et al., 2019). Dihydrotanshinone has shown cancer-preventive effects against liver cancer in different cell lines (HepG2 and HT-29 cell lines). It potentially reduces the levels of VEGF and Bcl2 while boosting the activity of caspases. These effects are involved in inhibiting angiogenesis and stimulating apoptosis by interfering with the intrinsic and extrinsic pathways of cell death (Su Y. S. et al., 2021).

β-carotene is a type of carotenoid obtained from different pigmented sources, like carrots. Carrot is a root vegetable called *Daucus carota* in scientific terms and belongs to the family Apiaceae (Metibemu et al., 2021). β-carotene has increased the number of apoptotic cells with an increase in the activity of caspases in colon cancer cells. It has also been reported to reduce the levels of NF-κB, Bcl2, and SOD proteins against colon cancer cell lines (HCT116 and SW480 cell lines) (Jain et al., 2018).

Artemisinin, derived from the shrubs of *Artemisia annua*, exhibits promising anticancer properties, particularly in breast cancer. At a dose of 300 mg/kg, it has demonstrated the remarkable ability to induce membranous translocation of beta-catenin while inhibiting the uncontrolled activation of the Wnt/b-catenin pathway, which is a critical signaling cascade implicated in cancer progression (Li et al., 2007). This dual mechanism effectively prevents cancer development and progression. The efficacy of artemisinin was evaluated in a preclinical study using Balb/c female nude mice as a model for breast cancer (Augustin et al., 2020). The results underscore its potential as a therapeutic agent in combating breast cancer, offering a glimpse into its mechanism of action and providing valuable insights for further clinical exploration (Ma et al., 2021).

Eugenol, a naturally occurring phenylpropanoid, is found in a variety of plants, including clove buds, tulsi leaves, turmeric, pepper, and ginger (Wang et al., 2020) Eugenol has been reported to reduce the activities of caspases and Bcl2, as shown on the MCF-7 cell line of breast cancer (do Nascimento Silva et al., 2016). Eugenol has reduced the tumor volume and proliferation of cancerous cells, both in combination with gemcitabine and alone (Li Z. et al., 2020). A US patent has also been published for the cancer-preventing profile of eugenol with the application number US20020103174A1. It has reduced tumor growth and size by more than 36% in the LNCaP and DU145 cell lines of human prostate cancer. Furthermore, *in vivo* activity has been performed using LNCaP human prostate tumor xenografts and concluded with a decreased tumor size and weight with increased apoptotic cells (Hussain et al., 2011).

Fennel seeds are obtained from the plant *Foeniculum vulgare* belonging to the family Umbelliferae. Since ancient times, the dried seeds have been used in food and medicine. Anethole has reduced the activity of NF-κB and STATs in female Sprague Dawley rats, leading to a decrease in tumor growth and proliferation in a breast cancer model (Lubet et al., 1997). Anethole has affected the TNF-α and JAK-STAT pathways of cancer development and showed cancer-preventive activity. Anethole has also been reported for its synergistic effects with curcumin (Contant et al., 2021).

### 3.3 Role of alkaloids

Alkaloids are any of a class of naturally occurring organic nitrogen-containing bases. Alkaloids have diverse and important physiological effects on humans and other animals. Well-known alkaloids include capsaicin, strychnine, sanguinarine, ephedrine, and nicotine. Alkaloids are significant chemical substances that provide a wealth of potential therapeutic targets and cancer-preventive potential as well. An important alkaloid, capsaicin is also known as 8-methyl-N-vanillyl-6-non amid and is obtained from *Capsicum annuum* (chili peppers) and *Capsicum frutescens* (includes bird’s eye chili) belonging to the family Solanaceae (Hayman and Kam, 2008). Capsaicin and its derivatives have been reported to overexpress SOCS to decrease STAT actions in gastric cancer (AGS cells) (Abdelrahman Mohamed and Abdullah AlQarni, 2019). It has also been reported to reduce NF-κB, resulting in cancer proliferation (Lubet et al., 1997). Another important alkaloid included in cancer prevention is sanguinarine, which is a polycyclic quaternary alkaloid extracted from bloodroot or *Sanguinaria canadensis* (Akhtar et al., 2019). In cancer prevention, sanguinarine has been found to reduce proliferation by reducing NF-κB levels (Zhang et al., 2019; Khan et al., 2021). It has also increased the level of BAX/BAK, which is responsible for the increase in apoptosis and necrosis in A431 and NHEK cells of epidermoid carcinoma (Achkar et al., 2017; Almeida et al., 2017; Kuttikrishnan et al., 2019). It has also shown synergistic effects with curcumin and quercetin (Almeida et al., 2017).

### 3.4 Role of phenolics

The wide presence of phenolic compounds in plants and herbal species (such as fruits, vegetables, cereals, olives, legumes, and cocoa) and beverages (such as tea, coffee, beer, and wine) contributes to the overall organoleptic characteristics of plant foods. Phenolics can boost the body’s immune system’s ability to identify and eradicate cancer cells and block angiogenesis, the formation of new blood vessels required for tumor development. EGCG, gingerol, fisetin, resveratrol, etc., are few examples in this class. Resveratrol is a type of polyphenolic compound isolated from grapes and their extract. It consists of the ripe fruits of *Vitis vinifera* belonging to the group of grapes (Huminiecki and Horbańczuk, 2018). Resveratrol decreases proliferation by targeting the NF-κB pathway (Mizerska-Kowalska et al., 2022). It has potentially activated apoptosis (reduced Bcl2 and Stat3) and activated the overexpression of SOCS, hence targeting the JAK-STAT pathway in the SCCHN cells of squamous cell carcinoma (Baek et al., 2016). It has also shown synergistic effects with 5-fluorouracil (a cytotoxic drug). An Indian patent office search clarifies that a patent has not been applied for the cancer-preventive action of the same drug, although the extract of *Vitis vinifera* has been granted a patent for a similar activity.

Another phenolic compound with cancer-prevention activity is EGCG. EGCG, an ester of epigallocatechin and gallic acid, is obtained from the plant *Camellia sinensis* belonging to the family Theaceae (Sojoodi et al., 2020). In terms of cancer-preventive activities, EGCG has been reported to reduce NF-ΚB overexpression and STAT actions (Rady et al., 2018; Ferrari et al., 2022). It has been reported to decrease VEGF, BAX, NF-κB, mitogen-activated extracellular signal-regulated kinase (MEK), and CDKs in HSC-3 cell lines of oral carcinoma (Aggarwal et al., 2022). EGCG has also shown synergistic effects with curcumin and resveratrol (Rady et al., 2018; Ferrari et al., 2022).

Gingerol is a polyphenolic compound with diverse activities. Gingerol has been extracted from *Zingiber officinale* belonging to the family Zingiberaceae. Gingerol has been reported to increase the expression of APC, p53, and TUNEL-positive nuclei and, subsequently, decrease the expression of TNF-α, IL-1β, inducible nitric oxide synthase (iNOS), COX-2, and cyclin D1 in male BALB/c mice with colorectal cancer (Farombi et al., 2020). There is an increase in the activity of gingerol when administered with corn oil with synergism. Gingerol has been reported to reduce tumor growth to a significant extent by targeting VEGF-A and WNT-3A. Gingerol has been shown to reduce p53 and NF-κB expression.

Another phenolic compound in this class is CAPE. Caffeic acid phenethyl ester (CAPE) is a natural phenolic chemical compound and an ester of caffeic acid and phenethyl alcohol (Kabała-Dzik et al., 2018). CAPE has also shown a cancer-preventive role by targeting the NF-κB activation pathway and STAT expressions in male Wistar rats with liver cancer (Carrasco-Legleu et al., 2004). It has been reported to reduce gene expression, resulting in the reduced proliferation of liver cancer cells.

Chemical compounds known as organometallic compounds (OMCs) have at least one bond between a metallic element and an organic molecule’s carbon atom. They are promising therapeutic candidates for the treatment of cancer because of their structural variety, ligand exchange, redox, and catalytic characteristics. OMCs include, for example, allicin and broccoli. Allicin is a diallylthiosulfinate or a type of organosulfur compound obtained from bulbs of *Allium sativum* (garlic) belonging to the family Amaryllidaceae (Chen et al., 2018). These organosulfur compounds have been found to be active against VEGF and proliferation by reducing the NF-κB activity in A/J mice for treating colorectal cancer (Saraswati and Agrawal, 2013). Garlic has also been reported to reduce the levels of iNOS expression in colorectal cancerous cells (Mansingh et al., 2018).

Broccoli comes under the group of cabbages belonging to the family Brassicaceae. Broccoli has been used to control cholesterol and fibromyalgia and in the treatment of several types of cancers (Tokuşoğlu and Parvizi, 2018). Metallic components of broccoli are active against cell proliferation, decrease NF-κB, and reduce the tumor size in SUM149 and SUM159 cell lines of breast cancer. It has been reported to inhibit CDKs in breast cancer cell lines (Burnett et al., 2017). It has also shown synergistic effects with curcumin, caffeic acid, and sorafenib. A patent office search clarifies that a patent has been filed for broccoli and its constituents for the treatment of cancer, but there is no filed patent for its cancer-preventive action.

### 3.5 Others

Vitamin D is a type of fat-soluble steroid that is essential for the body for its various pharmacological applications. The kinetic form of vitamin D is calcitriol (Rinninella et al., 2022). This sub-type of vitamin D has been reported to activate the expression of cleaved caspase-3, Bax, and p-AMPK and inhibit the expressions of p-Bcl-2, c-Myc, p-IGF-IR, p-mTOR, p-P70S6K, and p-S6, which further leads to apoptosis of the cancerous cells in Hs695T for amelanotic melanoma (Aslam et al., 2021). It has also shown synergistic effects with vitamin A and metformin. A patent office search clarifies that a patent has been filed for the chemo-preventive potential of vitamin D3 in WIPO PCT with the application number WO2006039281A2. It has been reported to induce cell cycle arrest at the G1 phase, causing decreased cell proliferation.

*Syzygium samarangense* (SS) is a type of flowering plant belonging to the family Myrtaceae. This extract of *Syzygium samarangense* has been reported to activate apoptosis and arrest the cell cycle in HEP G2-C8 cell lines of liver cancer (Su Z. Y. et al., 2021). This drug has elevated the levels of Bcl-2 and p53 for inducing apoptotic activity (Scuto et al., 2021). p53 also causes the secretion of PUMA and increases the rate of the intrinsic pathway of apoptosis.

#### 3.5.1 Toxicological impact of the chemo-preventive agents

Herbal cancer-preventive agents require their administration for a longer duration in minimum doses. So, it is important to consider their toxicological impact on the human body because these drugs interact with the signaling pathways of cells at the molecular level. Some herbal cancer-preventive agents may have toxicological effects on the body, particularly if taken in high doses or for prolonged periods of time. For example, some herbal agents, such as comfrey, may contain pyrrolizidine alkaloids that can cause liver damage and even cancer with long-term use. Their short mechanism of action in chemoprevention and reported toxicities are summarized in Table 2.

**TABLE 2:** Crosstalk in mechanistic approaches of cancer-preventive agents with reported toxicology.

Cancer-preventive phytomolecule	MOA involved in chemoprevention	Reported toxicity on prolonged use	Manifestation	Reference
Vitamin D3	Increases p53, caspase, and cytochrome and decreases proliferation, activating BAX	Reduces superoxide dismutase (SOD) levels	Impaired angiogenesis; a precognitive agent for various vascular diseases such as atherosclerosis. Elevated oxidative stress may be a major point of concern	Fukai and Ushio-Fukai (2011); Bakhtiari-Dovvombaygi et al. (2021)
Sulforaphane	Exhibits anti-proliferation, decreases NF-kB, reduces tumor size, and inhibits CDK	Synergistically boosts the overexpression of CEP55 and downregulates MMP28 with antimetabolites and NSAIDs	Exhibits increased concentrations of CEP55 and increases cell division as it is responsible for genomic instability; the deactivation of the MMP28 protein may create problems in wound healing or vein formation in pregnant women	Jeffery et al. (2015); Bozic et al. (2022)
Curcumin	Increases autophagy, caspase, cell death, and BAX and decreases Bcl2, NF-kB, and NRF-2	Reduces the activity of CYP1A1. (enhances the metabolism of estrogen)	Prolonged use of curcumin in higher doses reduces the levels of estrogen in both sexes. Reduced levels of CYP1A1 may cause the accumulation of steroids/fats and lead to CVDs and respiratory diseases	Nayak and Sashidhar (2010); Elkashty (2020)
Silymarin	Inhibits TNF-α and reduces tumor formation	Reduces MDA levels	Decreased MDA levels are beneficial in reducing ROS levels, creating difficulty in the diagnosis of other diseases such as psychiatry, chronic obstructive pulmonary disease, asthma, or cardiovascular diseases	Prakash Maurya et al. (2021); Ahmed et al. (2022)
Caffeic acid phenethyl ester	Inhibits NF-kB and reduces gene expression	Upregulates miR-182–5p on prolonged use	Impaired DNA repair with subsequent effects on the cell cycle, apoptosis, or genetic stability, leading to tumorigenesis. It also acts as a biomarker in cancer diagnosis	Krishnan et al. (2013); Hao et al. (2021)
Sanguinarine	Increases apoptosis and necrosis in cancerous cells	Causes severe cardiotoxicity	Severe malformation affecting heart rate, red blood cell number, blood flow dynamics, and stroke volume. Increased apoptosis may be a serious concern to cause heart-related complication, e.g., myocardial infraction	Wang et al. (2022)
Anethole	Decreases tumor incidence and multiplicity	Acetylcholinesterase activity	Induces rapid hydrolysis of acetylcholine neurotransmitter and may cause headache and drowsiness	Wang et al. (2021)
Resveratrol	Decreases proliferation, mRNA-EP, and STAT3 and increases SOCS	Deactivates inflammatory cytokines	Affects immunological reactions of body as it reduces the protective phenomenon of the human body against physical/chemical or mechanical stress	Hao et al. (2022)
Capsaicin	Blocks TNFa and decreases NFkB	Long-term ingestion of high amount of capsaicin causes gastrointestinal discomfort	Causes GI irritation, affecting oxidative stress and tissue permeability of the gastrointestinal tract, and leads to the development of irritable bowel syndrome	Xiang et al. (2021)
EGCG	Prevention and angiogenesis. Decreases VEGF and NF-kB and simultaneously increases BAX secretion	Interaction with PSMB5 results in the inhibition of proteasomes and their complexes	Inhibits the desirable actions of proteosomes and dysregulation of cell cycle progression and leads to prostate and other cancers	Cai et al. (2021)
6-Gingerol	Decreases NF-kB, CDK, and Bcl-2 and increases caspase	Reduces homocysteine and MDA levels	Decreased MDA levels are beneficial in reducing ROS levels; creates difficulty in diagnosis of other diseases such as psychiatry, chronic obstructive pulmonary disease, asthma, or cardiovascular diseases	Li et al. (2021)
Eugenol	Decreases Bcl2, activating caspase 3	Long-term use of eugenol causes a reduction in the secretion of the growth factor	Interaction with interleukins may cause such problems	Fuentes et al. (2022)
Allicin	Anti-angiogenesis and anti-proliferative	Hypotension and hyponatremia	By inhibiting angiotensin, it causes excessive diuresis	Dutta et al. (2021)
B-carotene	Increases caspase and BAX and decreases Bcl2, NF-kB, and NRF-2	Reduces MDA levels	Diagnosis of COPD or CVD may be critical as MDA is used as a biomarker for their diagnosis	Şahin and Karatepe (2022)
Dihydrotanshinone	Prevention: decreases CDKL1 and Bcl-2 and increases caspaseb 3 and mRNA expression	Inhibition of CYP2J2 may be helpful in chemoprevention	CYP2J2 provides protection for heart. Its decreased levels cause CVDs	Xu et al. (2018)
Fisetin	Inhibits cellular targets and proliferation	Activates heme oxygenase-1, which is responsible for oxidative cleavage of heme groups, leading to the generation of biliverdin and carbon monoxide and release of ferrous iron	Leads to jaundice and joint pain	Tsai et al. (2018)

## 4 Conclusion

**FIGURE 9:**
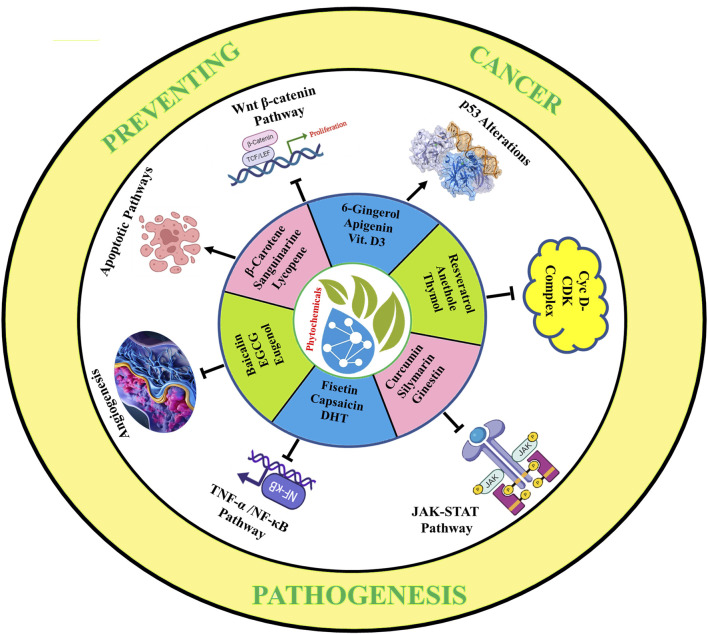
Impact of phyto-analogs in preventing cancer pathogenesis.

Cancer-prevention pathways involve a complex interplay of genetic, environmental, and lifestyle factors that can impact the development and progression of cancer. Although there is no sure-fire way to prevent cancer, there are many strategies that can be adopted to reduce the risk of developing the disease. Some of the most effective cancer-prevention strategies include maintaining a healthy diet and weight, getting regular exercise, avoiding tobacco and excessive alcohol consumption, and protecting oneself from harmful environmental exposures. The majority of the cancer-preventive phyto-analogs are included in a human’s daily diet in crude form, but there is a challenge in ensuring the minimum amount of required API to be taken for cancer-preventive actions. Additionally, regular cancer screening can help detect the disease at an early stage, when it is more treatable. Research has also identified several biological pathways and mechanisms that can impact cancer development and progression. These include oxidative stress, inflammation, DNA damage and repair, immune system function, and signaling pathways. Targeting these pathways with dietary products provides extraordinary evidence for preventing and targeting cancer in the future, as depicted in Figure 9. Numerous novel targets have been identified, including SOCS, STAT3, Nrf2, NF-B, cell cycle regulators cyclin D1, D2, and D3, INPs, and Wnt, which are expressed improperly in pre-cancer lesions. Worldwide, the scientific fraternity is involved in researching such newer drug candidates consistently for targeting these proteins at the molecular level to diminish the cancer progression and development further.

## Figures and Tables

**Table:** 

**WHO**	World Health Organization
**GCO**	Global Cancer Observatory
**JAK**	Janus kinase
**STAT**	Signal transducer and activator of transcription
**CRs**	Cytokine receptors
**DNA**	Deoxyribose nucleic acid
**HP1alpha**	SUMOylated heterochromatin protein 1-alpha
**SOCS**	Suppressor of cytokine signaling
**LRP**	Lipoprotein receptor-related protein
**Wnt**	Wingless-related integration site
**DVI**	Disheveled protein
**APC**	Adenomatosis polyposis coli
**CK**	Casein kinase
**GSK**	Glycogen synthase kinase
**ETS**	Erythroblast transformation-specific
**VEGF**	Vascular endothelial growth factor
**CDK**	Cyclin-dependent kinase
**EMT**	Epithelial–mesenchymal transition
**ZEB1 and ZEB2**	Zinc finger E-box-binding homeobox 1 and 2
**TNF-α**	Tumor necrosis factor-α
**SODD**	Silencer of the death domain
**TNFR**	Tumor necrosis factor receptor
**TRADD**	TNF-R1-associated death domain protein
**FADD**	FAS-associated death domain
**NF-κB**	Nuclear factor- κB
**cIAPs**	Cellular inhibitor of apoptosis protein-1
**iNOS**	Inducible nitric oxide synthase
**RIP1**	Receptor-interacting protein
**ROS**	Reactive oxygen species
**ATM**	Ataxia-telangiectasia-mutated
**PUMA**	p53-upregulated modulator of apoptosis
**Bcl-2**	B-cell lymphoma-2
**BAX**	Associated X protein
**VDAC**	Voltage-dependent anion-selective channel
**APAF-1**	Apoptosis protease-activating factor 1
**PDGF**	Platelet-derived growth factor
**PDGFR**	PDGF receptor
**Ang1/2**	Angiopoietin 1/2
**ECs**	Endothelial cells
**EPCs**	Endothelial progenitor cells
